# Cardiac Neurosis: The Psychological Impact of Self-Perceived Heart Disease

**DOI:** 10.7759/cureus.83233

**Published:** 2025-04-30

**Authors:** Shahbaz Khattak Haroon Ur Rashid, Samar Imran, Mariya Abbasi, Marium Nadeem Khan, Syed Muhammad Hassan Raza, Ali Sanan Ahmed, Muhammad Shahreyar, Fathima Farzana, Anaum Abbasi, Zainab Salahuddin, Anoosh Fatima, Ali Ahmad Abbasi

**Affiliations:** 1 Department of Medicine, St George's University Hospital NHS Trust, London, GBR; 2 Department of Internal Medicine, Shifa Tameer-E-Millat University, Islamabad, PAK; 3 Department of Medicine, Foundation University Medical College, Islamabad, PAK; 4 Department of Medicine, University of Toronto, Toronto, CAN; 5 Department of Medicine, Shifa College of Medicine, Islamabad, PAK; 6 Department of Internal Medicine, Dow International Medical College, Karachi, PAK; 7 Department of Medicine, GSM Medical Centre, Dubai, ARE; 8 Department of Medicine, University of Health Sciences Antigua, Piccadilly, ATG; 9 Department of Psychology, Brain Tech Clinic and Research Center, Islamabad, PAK

**Keywords:** aging, and quality of life, cardiac anxiety, cardiac neurosis, health anxiety, marital status, psychological distress, somatic symptoms

## Abstract

Introduction

Cardiac neurosis, also termed cardiophobia or heart anxiety, is characterized by persistent fear and misinterpretation of normal cardiac sensations as symptoms of heart disease, despite the absence of clinical evidence. This psychosomatic condition is associated with significant anxiety, depression, and somatic complaints, often leading to unnecessary medical consultations. The current study explored the psychological impact of self-perceived heart disease, emphasizing the relationship between cardiac-focused anxiety, generalized anxiety, and lifestyle disruptions in individuals without a verified cardiac diagnosis.

Methods

A cross-sectional, quantitative study design was employed with 100 adults aged 45 and above who reported concerns about heart health. Data were collected using standardized tools such as the Cardiac Anxiety Questionnaire (CAQ), Generalized Anxiety Disorder-7 (GAD-7), and SF-36 Health Survey. Participants completed a structured questionnaire via Google Forms. Analyses were conducted using IBM SPSS Version 26 (IBM Corp., Armonk, NY), utilizing correlation, ANOVA, and independent t-tests, with p-values <0.05 considered significant.

Results

The results demonstrated a significant positive correlation between generalized anxiety and cardiac anxiety (r = 0.595, p < 0.001), as well as between cardiac anxiety and physical health-related role limitations (r = 0.555, p < 0.001). These findings suggest that individuals with higher anxiety are more likely to perceive physical constraints associated with heart-related symptoms. The age-wise comparison revealed that participants aged 76 and above reported the highest levels of generalized anxiety (M = 16.11, SD = 4.83) and cardiac anxiety (M = 45.22, SD = 6.36), both with statistically significant group differences (F(3,96) = 7.48, p < 0.05; F(3,96) = 7.21, p < 0.05 respectively). Marital status also showed a notable effect: divorced individuals exhibited significantly higher generalized anxiety (M = 14.81, SD = 4.83) than single or married individuals (F(2,97) = 12.94, p < 0.05), along with elevated cardiac anxiety levels. These results collectively underscore the role of age and psychosocial context in shaping anxiety related to perceived heart health.

Conclusion

Cardiac neurosis is associated with a substantial psychological burden, including heightened anxiety, poor health perception, and reduced functionality. Despite the absence of cardiac pathology, individuals experience distress comparable to clinical cardiac patients. Early psychological screening and integrated mental health care are essential for effective management.

## Introduction

Cardiovascular diseases (CVDs) involve inflammation of coronary arteries that eventually results in the formation of atheroma, leading to functional deficiency as well as ultimately fatal situations in severe circumstances [1]. However, beyond organic heart diseases, cardiac neurosis presents a unique psychological condition where individuals experience persistent fear of having heart disease despite no medical diagnosis. Symptoms such as chest tightness, palpitations, and precordial discomfort are very similar to actual cardiac conditions but are caused mainly by anxiety [2]. Psychotherapy, cognitive behavioral therapy (CBT), and even conventional treatment such as herbal medicine or acupuncture have been suggested in some studies to relieve cardiac neurosis symptoms [3,4].

In recent years, increasing attention has been directed toward psychosomatic presentations within cardiology, particularly in individuals presenting with cardiac-related anxiety in the absence of confirmed pathology. In this study, we use the term "psychosomatic condition" to refer specifically to cardiac-focused health anxiety, which overlaps conceptually with somatoform and anxiety-related disorders. These individuals often report distressing somatic symptoms such as chest discomfort or palpitations without organic cardiac findings, reflecting heightened interoceptive awareness and maladaptive health-related beliefs. Understanding these presentations is crucial for early identification and management, particularly given their potential to mimic acute cardiac events and significantly impair daily functioning.

Furthermore, studies have found that somatic symptoms in cardiac neurosis patients closely resemble anxiety and depression, further supporting the necessity of psychological interventions in addition to medical reassurance [5]. Cardiac neurosis imitates heart disease, tending to result in excessive healthcare utilization, especially in females, the elderly, and inactive individuals [6].

Compared to patients with coronary heart disease, those with non-cardiac chest pain exhibit higher anxiety levels and distress, with negative life events acting as triggers [7]. Anxiety and depression have a bidirectional relationship with cardiovascular and psychiatric disorders, where each can make the other situation worse [8]. Those with depressive symptoms are at an increased risk for heart failure and myocardial infarction, pointing to the need for targeting interventions to reduce their CVD risk [9].

In this study, "perceived heart disease" refers to participants' self-reported worry or belief that they have heart problems without a confirmed medical diagnosis, assessed through structured demographic questions. "Health anxiety" is operationalized using the Generalized Anxiety Disorder-7 (GAD-7) scale, while "perception of bodily symptoms" is measured through the Cardiac Anxiety Questionnaire (CAQ), focusing on fear, attention to, and avoidance of cardiac-related sensations.

Furthermore, studies on atrial fibrillation patients show that anxiety and depression significantly impair quality of life, affecting both mental and physical health [10]. The incidence of psychological distress among cardiac patients is startlingly high, with more than 54% suffering from severe depression and 19.2% presenting with symptoms of severe anxiety. These are especially prevalent among females and those with post-traumatic stress disorder, low self-esteem, and poor social support [11]. Anxiety and depression are common in hypertensive and cardiac patients and are strongly associated with high stress, low support, unemployment, and unhealthy living [12]. Moreover, anxiety disorders frequently coexist with cardiovascular, respiratory, and gastrointestinal conditions, making differential diagnosis and treatment challenging [13]. Research indicates that moderate levels of depression (18%), anxiety (28%), and stress (13%) are prevalent among cardiac rehabilitation patients, negatively impacting treatment adherence. Anxiety and stress are strong predictors of depression, whereas depression and stress are key risk factors for anxiety [14]. The connection between anxiety, depression, and life satisfaction is also essential since increased levels of distress lead to lower quality of life as well as well-being [15].

Despite its wide-ranging influence, cardiac neurosis is still a poorly researched condition, especially in health-anxious and psychosomatically concerned populations. There is a great need to investigate how anxiety related to heart health influences perceived symptoms, changes in lifestyle, and psychological distress. The present study intends to identify the psychological impact of perceived heart disease in understanding the association among cardiac anxiety, perceived symptoms, and behavioral adjustments. Through an analysis of these interfaces, this study will be able to add to the knowledge of health anxiety and its effects, thereby necessitating the convergence of mental and physical healthcare strategies.

Rationale

Cardiac neurosis, also referred to as cardiophobia or heart-focused anxiety, is characterized by persistent apprehension and preoccupation with the possibility of developing heart disease despite the absence of any clinical diagnosis. Individuals often misinterpret benign bodily sensations such as increased heart rate or chest tightness as signs of severe cardiac conditions, leading to heightened distress and functional impairment. This cycle is reinforced by psychological mechanisms such as interoceptive awareness (heightened sensitivity to internal bodily sensations), attentional bias toward perceived cardiac symptoms, and misattribution of normal physiological responses to life-threatening conditions.

While CVD remains a leading global health concern, a significant but underrecognized population experiences cardiac-related psychological distress without organic pathology. These individuals frequently overuse healthcare services, pursue unnecessary diagnostic procedures, and alter their behavior based on fear rather than medical necessity. However, research addressing the specific cognitive-affective processes driving this phenomenon remains limited, especially in middle-aged and older populations who are at increased risk of both cardiac events and anxiety-related somatic symptoms.

This study addresses a critical gap by investigating the interplay between cardiac anxiety, generalized anxiety, and lifestyle disruption, aiming to clarify the underlying psychological mechanisms that sustain fear and avoidance behaviors. By identifying these patterns, the findings can inform screening practices in primary care and cardiology settings, offering guidance for timely referrals to mental health professionals. Ultimately, this research contributes to an integrated model of care that recognizes the psychological dimensions of somatic symptom presentations and supports early psychological intervention to prevent chronic functional impairment.

Objectives

This study aims to investigate the association between anxiety regarding heart health and perception of bodily symptoms. It also seeks to explore the influence of self-perceived heart disease on lifestyle factors such as everyday functioning, exercise, and health behaviors.

## Materials and methods

Study design and setting

This research employed a cross-sectional, quantitative survey design to investigate the psychological effects of self-reported heart disease. Participants answered self-report questionnaires measuring levels of anxiety, symptom awareness, and lifestyle changes associated with cardiac issues.

The research included adults aged 45 and above, who experienced recurring worries about the health of their heart, irrespective of an established medical diagnosis. Participants were also eligible if they had a history of heart-related problems, high cholesterol, or high blood pressure, and agreed to participate in the research. The study excluded those with clinically severe psychiatric illnesses that would impair participation.

Sample size and technique

The sample size calculated according to the WHO sample size was 100, with a confidence interval of 95%, an expected population proportion of 0.90, and an absolute precision of 0.05. A convenience sampling approach was used to collect data from 100 participants. Age groups were stratified into intervals (45-55, 56-65, 66-75, and 76+) based on both clinical considerations and sample distribution. The 76+ age group was treated as a distinct category due to elevated health vulnerability, cognitive decline risk, and differences in coping mechanisms observed in older populations. These categories also align with conventional groupings used in epidemiological studies and allow for a nuanced interpretation of age-related psychological effects. The population proportion of 0.90 was used based on prior literature, suggesting that a high percentage of individuals with cardiac-related concerns experience significant psychological distress, even in the absence of confirmed heart disease.

Data collection tools and procedure

Participants were recruited online through the distribution of a Google Forms link. The link was shared via email lists, community social media groups, and professional networks targeting individuals aged 45 and above with a history of heart-related concerns or ongoing worry about cardiac health. An initial eligibility screening was embedded in the form to confirm participants met the inclusion criteria before proceeding to the full survey. Information about the study purpose, confidentiality, and voluntary participation was presented on the first page, and informed consent was obtained electronically before participants could access the questionnaires. We used different questionnaires to assess cardiac anxiety, overall health-related quality of life, and anxiety levels. The CAQ was used to assess heart-focused anxiety, which measures fear of cardiac symptoms, avoidance behaviors, and reassurance-seeking tendencies. It comprises 18 items and can be subdivided into three scales: fear (8 items), avoidance (5 items), and attention (5 items). Each item is measured using a 5-point Likert scale, with scores ranging from 0 (never) to 4 (always). A higher score indicates more symptoms, a greater number of symptoms, or both. Reliability analysis of the 18-item CAQ revealed good internal consistency of the total and subscale scores [6] (Appendix). The SF-36 Health Survey Questionnaire was used to evaluate the overall health-related quality of life. The SF-36 is an instrument measuring eight health-related subscales: physical functioning (10 items), role limitations due to physical health (4 items), bodily pain (2 items), general health perception (5 items), vitality (4 items), social functioning (2 items), role limitations due to emotional problems (3 items), and perceived mental health (5 items). The Cronbach's alpha value for the SF-36 was recorded as 0.90, which showed high internal consistency reliability [16]. The GAD-7 Questionnaire was included to screen for symptoms of general anxiety. The GAD-7 is a self-reported scale with seven items aimed at assessing symptoms that define GAD. Each item is rated on a 4-point scale (0=not at all, 3=nearly every day). Total scores can range from 0 to 21, where higher scores indicate more severe GAD symptoms. The internal consistency of the GAD-7 was excellent (Cronbach alpha = 0.92). Test-retest reliability was also good (intraclass correlation = 0.83) [17], Structured questionnaire was administered to collect demographic information (age, gender, education, employment status, marital status, history of heart-related concerns, and mental health conditions). Google Forms were used for data collection.

The choice of assessment instruments was guided by their relevance and strong psychometric properties for capturing the study's core constructs. The CAQ specifically measures heart-focused anxiety, including fear of cardiac symptoms and reassurance-seeking behavior, making it directly applicable to our population of individuals concerned about heart health. The SF-36 was selected to capture the broader impact of these concerns on physical, emotional, and social functioning, enabling a comprehensive assessment of health-related quality of life. The GAD-7 complements these by measuring generalized anxiety symptoms, which are frequently comorbid with health anxiety. Together, these tools offer a multidimensional perspective on lifestyle disruption and psychological burden. Concern about heart health was operationalized using an intake screening question focusing on recurring worry or preoccupation with cardiac functioning in the absence of a confirmed medical diagnosis.

Informed consent was obtained before participation, and the consent form contained information on the study purpose, maintenance of confidentiality, and voluntary participation. The demographic questionnaire, CAQ, SF-36, and GAD-7 scales were completed in one session. The Institutional Review Board of Brain Wave Research Center approved it with IRB-2024-2396 before starting the research. Since each participant was indeed over the age of 18, the requirement for parental or guardian consent was omitted. During the study, the confidentiality of participant identities was maintained. The data collected were strictly for research purposes.

The analysis was conducted using IBM SPSS Statistics software Version 26 (IBM Corp., Armonk, NY, USA). The analytical and descriptive statistics were used with regression, chi-square, independent-sample t-test, mean, and standard deviation. A p-value of less than 0.05 was used as the level of significance.

## Results

Table 1 presents the descriptive statistics of the demographic variables, including gender, age, education, and marital status. The sample consists of 100 individuals. In terms of gender distribution, there were 41 (41.0%) males and 59 (59.0%) females. Regarding age, most participants fall within the age group of 56-65 years (47, 47.0%), followed by those aged 66-75 (31, 31.0%). A smaller proportion of participants were in the age range of 45-55 years (13, 13.0%), while the least represented category includes individuals aged 76 and above (9, 9.0%). In terms of education, most participants had completed graduation (78, 78.0%), 16 (16.0%) had an intermediate level of education, and only 6 (6.0%) had attained post-graduation. Marital status data indicated that most participants were married (62, 62.0%), 26 (26.0%) were divorced, and 12 (12.0%) were single.

**Table 1:** Descriptive statistics of demographic variables

Variable	Frequency	Percentage
Gender	-	-
Male	41	41.0%
Female	59	59.0%
Age	-	-
45-55	13	13.0%
56-65	47	47.0%
66-75	31	31.0%
76 above	9	9.0%
Education	-	-
Intermediate	16	16.0%
Graduation	78	78.0%
Post-graduation	6	6.0%
Marital status	-	-
Single	12	12.0%
Married	62	62.0%
Divorced	26	26.0%

Table 2 presents the intercorrelations among the study variables. Generalized anxiety showed a strong positive correlation with cardiac anxiety (r = 0.595, p < 0.001), role limitations due to physical health (r = 0.674, p < 0.001), and role limitations due to emotional problems (r = 0.644, p < 0.001). Generalized anxiety also correlated significantly but moderately with overall health (r = 0.378, p < 0.001) and mental health component (r = 0.373, p < 0.001), and showed modest but significant relationships with social functioning (r = 0.285, p < 0.05) and vitality (r = 0.285, p < 0.05). However, generalized anxiety was negatively correlated with general health perception (r = -0.500, p < 0.001) and physical functioning (r = -0.396, p < 0.001), suggesting that higher anxiety is associated with poorer perceived general health and decreased physical capability. Cardiac anxiety strongly correlated with role limitations due to physical health (r = 0.555, p < 0.001) and emotional problems (r = 0.539, p < 0.001), and had moderate negative correlations with general health perception (r = -0.498, p < 0.001). Cardiac anxiety was moderately positively correlated with social functioning (r = 0.397, p < 0.001) and bodily pain (r = 0.298, p < 0.05) and showed a weaker positive relationship with overall health (r = 0.278, p < 0.05) and physical health component (r = 0.200, p < 0.05). General health perception negatively correlated significantly with role limitations due to emotional problems (r = -0.422, p < 0.001), role limitations due to physical health (r = -0.371, p < 0.001), bodily pain (r = -0.316, p < 0.001), and overall health (r = -0.380, p < 0.001). Physical functioning was negatively associated with role limitations due to physical health (r = -0.600, p < 0.001) and emotional problems (r = -0.515, p < 0.001) but strongly positively correlated with the physical health component (r = 0.754, p < 0.001). Role limitations due to physical health and emotional problems exhibited a very strong positive correlation with each other (r = 0.868, p < 0.001), indicating significant overlap between these two variables. Social functioning was positively correlated with bodily pain (r = 0.239, p < 0.05), overall health showed moderate positive correlations with vitality (r = 0.372, p < 0.001), and vitality correlated positively with the mental health component (r = 0.969, p < 0.001) and mental health variable itself (r = 0.592, p < 0.001). Lastly, mental health component scores strongly correlated with vitality (r = 0.969, p < 0.001) and mental health scores (r = 0.695, p < 0.001). These findings suggest that anxiety measures, both generalized and cardiac-specific, are significantly associated with reduced physical and emotional functioning, impaired general health perception, and lower vitality. High correlations between role limitations due to physical and emotional problems reflect their shared influence on overall health outcomes.

**Table 2:** Intercorrelation between study variables **p<0.001, *p<0.05, considered significant

Variables	Generalized anxiety	Cardiac anxiety	General health perception	Physical functioning	Role limitations due to physical health	Role limitations due to emotional problems	Social Functioning	Body Pain	Vitality	Mental Health	Overall, Health	Physical Health Component	Mental Health Component
Generalized anxiety	-	-	-	-	-	-	-	-	-	-	-	-	-
Cardiac anxiety	0.595**	-	-	-	-	-	-	-	-	-	-	-	-
General health perception	-0.500**	-0.498**	-	-	-	-	-	-	-	-	-	-	-
Physical functioning	-0.396**	-0.187	0.126	-	-	-	-	-	-	-	-	-	-
Role limitations due to physical health	0.674**	0.555**	-0.371**	-0.600**	-	-	-	-	-	-	-	-	-
Role limitations due to emotional problems	0.644**	0.539**	-0.422**	-0.515**	0.868**	-	-	-	-	-	-	-	-
Social functioning	0.285*	0.397**	-0.234*	0.143	0.167	0.145	-	-	-	-	-	-	-
Bodily pain	0.193	0.298*	-0.316**	-0.019	0.083	0.102	0.239*	-	-	-	-	-	-
Vitality	0.285*	0.091	-0.207*	-0.012	0.160	0.263**	0.159	-0.160*	-	-	-	-	-
Mental health	0.069	-0.070	-0.050	0.186	-0.151	-0.107	0.130	-0.155	0.592**		-	-	-
Overall health	0.378**	0.278*	-0.380**	-0.127	0.262**	0.257**	0.182	0.137	0.372**	0.262**	-	-	-
Physical health component	0.033	0.200*	-0.249*	0.754**	-0.150	-0.103	0.349**	0.398**	0.133	0.190	0.400**	-	-
Mental health component	0.373**	0.184	-0.266**	-0.036	0.249*	0.359**	0.280**	-0.119	0.969**	0.695**	0.410**	0.166	-

Table 3 shows the comparison between males and females. Overall, there were no statistically significant gender differences across any of the measured outcomes. Specifically, generalized anxiety and cardiac anxiety showed similar levels for both male and female participants, suggesting that anxiety experiences were comparable between genders in this sample. General health perceptions were also similar, indicating that both genders had comparable self-assessments of their health status. Regarding physical functioning, males and females showed equivalent functional capabilities, implying that physical health and functional activities were not significantly different based on gender. Similarly, although females reported slightly higher role limitations due to physical and emotional health problems, these differences did not reach statistical significance, suggesting that both genders experience similar impacts of physical and emotional conditions on daily functioning. Social functioning and bodily pain were also comparable between males and females, indicating that social interaction capabilities and experiences of bodily pain did not vary meaningfully by gender. The similarity in vitality scores further suggests that male and female participants experienced comparable levels of energy and fatigue. Mental health scores and overall health assessments also revealed no significant gender differences, demonstrating that emotional well-being and overall health evaluations were equally distributed between genders. Finally, both physical and mental health components, which reflect broader dimensions of health status, were also similar for males and females, further reinforcing the absence of meaningful gender disparities in this study. The Cohen’s d effect sizes were consistently small across all variables, highlighting that any minor differences observed between genders were practically negligible. Thus, gender did not appear to be a significant factor influencing psychological or health-related outcomes in this sample.

**Table 3:** Comparison of gender across study variables using independent sample t-tests M, mean; SD, standard deviation; LL, lower limit; UL, upper limit; CI, confidence interval

Variables	Male		Female		t(98)	p	95% CI		Cohen’s d
	M	SD	M	SD			LL	UL	
Generalized anxiety	10.46	4.72	10.93	5.67	-0.435	0.665	-2.61	1.67	-0.09
Cardiac anxiety	37.2	10.1	38.86	10.57	-0.791	0.431	-5.86	2.52	-0.16
General health perception	2.17	0.83	2.02	0.84	0.903	0.369	-0.18	0.49	0.18
Physical functioning	27.46	4.87	26.37	4.82	1.108	0.271	-0.86	3.04	0.23
Role limitations due to physical health	1.29	1.4	1.71	1.62	-1.344	0.182	-1.04	0.2	-0.27
Role limitations due to emotional problems	0.95	1.16	1.24	1.32	-1.12	0.265	-0.79	0.22	-0.23
Social functioning	3.22	0.69	3.19	0.8	0.215	0.83	-0.27	0.34	0.04
Body pain	4.9	1.53	4.98	1.71	-0.242	0.809	-0.74	0.58	-0.05
Vitality	22.12	6.19	21.98	5.21	0.121	0.904	-2.13	2.41	0.02
Mental health	5.27	1.91	5.17	1.81	0.262	0.794	-0.65	0.85	0.05
Overall, health	8.15	1.99	8.41	1.88	-0.665	0.507	-1.04	0.52	-0.14
Physical health component	41.8	4.55	41.47	5.07	0.334	0.739	-1.63	2.29	0.07
Mental health component	31.56	7.55	31.58	7.31	-0.01	0.992	-3.0	2.97	-0.0

Table 4 presents the descriptive statistics and ANOVA results comparing health and psychological outcomes across four age groups (45-55, 56-65, 66-75, and 76+ years). The analysis reveals significant age-related differences in multiple domains. Notably, generalized anxiety and cardiac anxiety levels increase with age, with the highest scores observed in the 76+ age group. These differences are statistically significant (p < 0.05) and associated with large effect sizes (η² = 0.19 and 0.18, respectively), indicating that age accounts for a substantial portion of the variance in anxiety symptoms. Similarly, role limitations due to physical health and role limitations due to emotional problems showed significant differences between groups, both with large effect sizes (η² = 0.19 and 0.22). These limitations are more pronounced in older adults, especially those aged 66 and above, suggesting increased functional and emotional challenges with advancing age. General health perception, physical functioning, and overall health also vary significantly by age, with moderate effect sizes (η² = 0.10 to 0.14). Specifically, physical functioning tends to decline with age, while self-perceived general health diminishes gradually. These findings highlight the progressive nature of physical and perceptual health changes across the lifespan. In contrast, measures such as social functioning, bodily pain, vitality, mental health, physical health component, and mental health component do not differ significantly across age groups (p > 0.05), and effect sizes remain small (η² < 0.06). This suggests relative stability in these areas or that age may not be the primary factor influencing these outcomes. Overall, the results indicate that older age is significantly associated with higher levels of anxiety and greater role limitations, both physically and emotionally, while certain health perceptions and physical functioning also show age-related trends. Other aspects of well-being remain relatively consistent, underscoring the multidimensional impact of aging on health.

**Table 4:** Descriptive statistics and ANOVA results by age group SD, standard deviation; η², effect size *p<0.05

Measure	45-55, mean (±SD)	56-65, mean (±SD)	66-75, mean (±SD)	76+, mean (±SD)	η²	F(3,96)
Generalized anxiety	9.77 (±4.92)	8.87 (±4.31)	12.42 (±5.50)	16.11 (±4.83)	0.19	7.48*
Cardiac anxiety	37.15 (±10.83)	34.02 (±10.27)	42.87 (±8.36)	45.22 (±6.36)	0.18	7.21*
General health perception	2.08 (±0.86)	2.36 (±0.76)	1.81 (±0.83)	1.56 (±0.73)	0.12	4.48*
Physical functioning	26.54 (±7.05)	28.28 (±4.72)	25.58 (±3.44)	23.89 (±3.95)	0.10	3.44*
Role limitations due to physical health	1.38 (±1.39)	0.91 (±1.32)	2.26 (±1.55)	2.56 (±1.42)	0.19	7.37*
Role limitations due to emotional problems	0.85 (±1.14)	0.60 (±1.01)	1.84 (±1.24)	1.78 (±1.30)	0.22	8.81*
Social functioning	3.31 (±0.63)	3.11 (±0.81)	3.26 (±0.77)	3.33 (±0.50)	0.01	0.48
Bodily pain	5.62 (±1.45)	4.57 (±1.85)	5.19 (±1.22)	5.11 (±1.62)	0.06	1.86
Vitality	21.15 (±3.00)	21.66 (±6.17)	22.39 (±5.57)	24.11 (±5.71)	0.02	0.62
Mental health	4.46 (±1.61)	5.11 (±1.86)	5.35 (±1.85)	6.33 (±1.73)	0.06	2.00
Overall health	7.23 (±1.36)	7.94 (±1.62)	8.84 (±1.97)	9.89 (±2.57)	0.14	5.41*
Physical health component	40.77 (±7.30)	41.70 (±5.11)	41.87 (±2.99)	41.44 (±4.98)	0.01	0.17
Mental health component	29.77 (±4.78)	30.47 (±7.84)	32.84 (±7.15)	35.56 (±7.52)	0.05	1.84

Table 5 illustrates the descriptive statistics and ANOVA results comparing health and psychological outcomes across different education levels (intermediate, graduation, and post-graduation). A significant difference was found in generalized anxiety, where individuals with post-graduate education reported the highest levels of anxiety (M = 17.83), followed by those with intermediate (M = 12.19) and graduation-level education (M = 9.90). This difference was statistically significant (F(2,97) = 8.00, p < 0.05) with a large effect size (η² = 0.19), indicating that education level substantially influences generalized anxiety levels. Role limitations due to physical health and role limitations due to emotional problems also varied significantly across education groups (p < 0.05), with the highest limitations reported among those with post-graduate education. Both variables showed large effect sizes (η² = 0.19 and 0.22, respectively), suggesting a strong relationship between higher educational attainment and greater role limitations. These findings may reflect heightened awareness or reporting of health-related limitations among those with more education. Other measures, such as cardiac anxiety, physical functioning, and overall health, approached significance but did not meet the threshold (p > 0.05). Still, moderate effect sizes (η² = 0.10-0.18) suggest potential educational influences worth further exploration. In contrast, variables such as social functioning, bodily pain, vitality, mental health, physical health component, and mental health component did not differ significantly between education groups, with small effect sizes (η² ≤ 0.06). This indicates that, for these domains, education level may not be a primary differentiating factor. In summary, the results suggest that education level is significantly associated with differences in anxiety and perceived role limitations, with higher educational attainment linked to greater reported psychological distress and functional limitations. Other health indicators appear less influenced by education in this sample.

**Table 5:** Descriptive statistics and ANOVA results by education level SD, standard deviation; η², effect size *p<0.05

Measure	Intermediate, mean (±SD)	Graduation, mean (±SD)	Post-graduation, mean (±SD)	F(2,97)	η²
Generalized anxiety	12.19 (±5.37)	9.90 (±4.97)	17.83 (±2.56)	8.00*	0.19
Cardiac anxiety	38.75 (±11.20)	37.33 (±10.15)	47.67 (±6.62)	2.91	0.18
General health perception	2.19 (±0.91)	2.10 (±0.82)	1.50 (±0.84)	1.62	0.12
Physical functioning	25.06 (±6.43)	27.40 (±4.46)	24.00 (±3.16)	2.71	0.10
Role limitations due to physical health	1.94 (±1.53)	1.31 (±1.47)	3.50 (±0.84)	7.04*	0.19
Role limitations due to emotional problems	1.56 (±1.09)	0.94 (±1.23)	2.33 (±1.21)	4.99*	0.22
Social functioning	3.19 (±0.40)	3.17 (±0.81)	3.67 (±0.52)	1.24	0.01
Bodily pain	4.69 (±1.25)	4.95 (±1.70)	5.67 (±1.63)	0.79	0.06
Vitality	23.63 (±4.60)	21.54 (±5.82)	24.33 (±4.23)	1.47	0.02
Mental health	4.56 (±1.82)	5.26 (±1.84)	6.33 (±1.51)	2.17	0.06
Overall health	8.19 (±2.48)	8.19 (±1.65)	10.00 (±2.97)	2.58	0.14
Physical health component	39.88 (±6.11)	41.85 (±4.63)	43.17 (±2.93)	1.44	0.01
Mental health component	32.94 (±5.90)	30.90 (±7.64)	36.67 (±5.39)	2.08	0.05

Table 6 presents the descriptive statistics and ANOVA results for psychological and health-related variables based on marital status (single, married, and divorced). The analysis indicates that generalized anxiety differs significantly across marital groups (F(2,97) = 12.94, p < 0.05), with divorced individuals reporting the highest levels (M = 14.81), compared to single (M = 9.33) and married individuals (M = 9.31). This effect is strong, as indicated by a large effect size (η² = 0.21). Similarly, cardiac anxiety is significantly elevated among divorced individuals (M = 42.92), followed by married (M = 37.32) and single (M = 32.33) participants, with a moderate effect size (η² = 0.10). Significant differences also appear in general health perception, role limitations due to physical health, role limitations due to emotional problems, and social functioning. Divorced individuals again report greater limitations and lower perceived health across these domains, and effect sizes range from η² = 0.08 to 0.12. These findings suggest a consistent pattern of poorer self-reported health and functional outcomes among divorced participants compared to their single or married counterparts. Although vitality, overall health, and mental health components did not reach conventional significance levels (p > 0.05), they showed emerging trends, particularly in the mental health component, where divorced individuals again report the highest scores (M = 34.65), with a small-to-moderate effect size (η² = 0.06). Variables such as physical functioning, bodily pain, mental health, and physical health component did not show statistically significant differences across marital status, with small effect sizes (η² ≤ 0.04), suggesting these areas are relatively stable regardless of marital status. In summary, the results indicate that marital status is associated with significant differences in anxiety, perceived role limitations, and social functioning, with divorced individuals consistently reporting worse outcomes. These patterns highlight the potential psychological and functional vulnerabilities associated with being divorced, while married individuals tend to report better outcomes across several domains.

**Table 6:** Descriptive statistics and ANOVA results by marital status SD, standard deviation; η², effect size *p<0.05

Measure	Single, mean (±SD)	Married, mean (±SD)	Divorced, mean (±SD)	F(2,97)	η²
Generalized anxiety	9.33 (±6.76)	9.31 (±4.23)	14.81 (±4.83)	12.94*	0.21
Cardiac anxiety	32.33 (±14.55)	37.32 (±9.46)	42.92 (±8.48)	5.26*	0.10
General health perception	2.42 (±1.08)	2.18 (±0.71)	1.69 (±0.88)	4.47*	0.08
Physical functioning	25.83 (±8.85)	27.56 (±3.92)	25.50 (±4.19)	1.98	0.04
Role limitations due to physical health	1.33 (±1.83)	1.21 (±1.38)	2.42 (±1.47)	6.45*	0.12
Role limitations due to emotional problems	0.83 (±1.34)	0.90 (±1.18)	1.77 (±1.21)	5.09*	0.10
Social functioning	2.67 (±1.30)	3.23 (±0.49)	3.38 (±0.85)	4.08*	0.08
Bodily pain	4.25 (±2.63)	5.02 (±1.34)	5.12 (±1.68)	1.30	0.03
Vitality	22.08 (±6.95)	21.19 (±5.35)	24.04 (±5.23)	2.43	0.05
Mental health	5.33 (±2.35)	5.08 (±1.81)	5.46 (±1.70)	0.42	0.01
Overall health	8.58 (±2.07)	8.00 (±1.67)	8.88 (±2.30)	2.14	0.04
Physical health component	40.00 (±9.51)	41.79 (±3.95)	41.92 (±3.69)	0.76	0.01
Mental health component	30.92 (±8.37)	30.40 (±7.21)	34.65 (±6.64)	3.24*	0.06

## Discussion

The present study sought to emphasize the psychological consequences of self-reported heart disease, also referred to as cardiac neurosis, on anxiety, depression, somatic symptoms, and cardiac-focused anxiety. Our results show a significant relationship between the perception of CVD and higher levels of anxiety, depression, and somatic symptoms. This supports the hypothesis that those individuals who believe they are suffering from heart disease, even in the absence of medical evidence, show psychological distress comparable to those with clinically diagnosed cardiac illness. These findings align with the previous research [15,18].

The present results are also consistent with earlier research that examined gender variation in psychological responses among cardiovascular patients. Gender had no significant effects on generalized anxiety, cardiac anxiety, general perception of health, or physical function in this experiment. Male and female participants had similar levels of distress on all these dimensions. These results echo those from studies involving patients with implantable cardioverter-defibrillators (ICDs), which also reported negligible gender differences in anxiety or overall quality of life, although women often reported lower physical functioning and vitality [19].

Contrary to some earlier studies, the current research identified a positive correlation between age and psychological distress, with participants aged 76 years and above exhibiting higher levels of both generalized and cardiac-focused anxiety. This result contradicts earlier evidence that indicated that younger patients generally reported more psychological distress, with such symptoms reducing as age increases [20]. This difference could be due to age-related variations in perceptions of health, coping, or access to support systems in non-clinically diagnosed samples.

Interestingly, the study also found that respondents with post-graduate education experienced more generalized anxiety and greater incapacities due to physical and emotional impairments. While previous research typically links lower education with higher levels of psychological distress, owing to socioeconomic deprivation and restricted access to mental health treatment [21], these findings provide a different account.

The observed correlation (r = 0.595) between cardiac anxiety and reduced quality of life is considered moderate to strong according to Cohen’s conventions, suggesting that individuals with higher heart-related anxiety experience significantly greater impairments in daily functioning. This finding supports the integration of psychological screening into routine medical care, particularly within cardiology and primary care settings. Given the overlap between somatic symptoms and anxiety disorders, professionals such as health psychologists, behavioral medicine specialists, and general practitioners should be engaged in early identification and referral processes. Such collaboration can improve patient outcomes and reduce unnecessary medical investigations.

The finding that psychological distress increased with age, particularly among participants aged 76 and above, contrasts with some prior studies suggesting that younger individuals typically report higher levels of distress. Several factors may account for this divergence. Age-related changes in health perception, including heightened vulnerability to illness and increased focus on bodily symptoms, could intensify anxiety among older adults, even without a clinical diagnosis. Coping strategies may also shift with age, with older adults potentially relying more on internalized worry rather than active help-seeking. Additionally, reductions in social support networks due to bereavement or social isolation may exacerbate distress. Possible cohort effects, such as differing life experiences and attitudes toward health between older and younger generations, may also influence symptom perception. Furthermore, differences in sample characteristics - specifically, the focus on a non-clinically diagnosed population in the current study versus clinical samples in earlier research - and variations in the measurement tools used may partly explain the discrepancy. Clarifying these factors highlights the need for age-sensitive screening and intervention approaches in future psychological and gerontological research on cardiac anxiety.

Marital status also appeared to be a relevant variable, with divorced patients showing the greatest degrees of generalized and cardiac anxiety, as well as lower social functioning, general health perception, and role fulfillment. These results are per current literature showing that divorced or recently separated individuals are more at risk of depression and anxiety [22].

The interpretation of marital status effects has been revised to reflect that no post hoc comparisons confirmed statistically significant differences between groups. Higher scores in social functioning and mental health domains indicate better outcomes, and divorced individuals did not consistently show worse functioning compared to others. Given the cross-sectional design, causality cannot be inferred. Psychosocial factors such as reduced support and increased stress after divorce may still contribute to vulnerability; however, further longitudinal research is needed to clarify these associations.

Caution is warranted in interpreting self-reported cardiac distress as equivalent to clinically diagnosed cardiac conditions, as no direct clinical verification was conducted. Gender comparisons showed non-significant differences across anxiety, health perception, and physical functioning. This finding could reflect measurement sensitivity limitations, sample size constraints, or cultural factors affecting symptom reporting, which may have minimized gender disparities.

Despite its strengths, the study has its limitations. The sample was limited and from only one clinical site, and thus there is limited generalizability. Self-report measures, although convenient, are also prone to bias and may not be fully representative of clinical complexity in participant experiences. In addition, this being a cross-sectional study hinders causal inferences. It is therefore unclear whether participants' psychological distress preceded their views of cardiac problems or vice versa.

Future studies should also consider longitudinal designs to clarify the temporal dynamics of cardiac neurosis. There is a dire need to conduct intervention-based studies into the effectiveness of psychotherapeutic approaches such as cognitive-behavior therapy or mindfulness-based interventions in alleviating such symptoms as those of cardiac-focused anxiety. Furthermore, investigating biological markers of stress and anxiety can help shed light on the interaction between physiological arousal and symptom perception.

Despite these limitations, this study contributes meaningfully by addressing an underexplored intersection between cardiac symptom perception and psychological distress in a non-clinically diagnosed population. A key strength lies in the incorporation of demographic variables such as age, education, and marital status to illuminate specific risk factors, offering nuanced insights that can inform early screening, patient education, and referral guidelines within primary care and cardiology contexts. By highlighting the moderating influence of psychosocial characteristics, the present study lays groundwork for more tailored and integrated mental health interventions targeting cardiac anxiety.

## Conclusions

This study draws attention to the intense psychological impact of cardiac neurosis, showing how perceived heart disease may lead to increased anxiety, depression, somatic symptoms, and cardiac-specific anxiety even when clinically proven cardiac pathology is not present. The findings suggest a chronic distress pattern related to functional impairment and health in individuals who perceive they have heart disease, implying a high psychosomatic component of symptom experience.

The results underscore the importance of early detection and psychological evaluation of individuals who complain of cardiac symptoms but lack objective medical evidence. Treating cognitive distortions, health anxiety, and emotional disturbance underlying cardiac neurosis using integrative strategies, such as psychoeducation, psychotherapy, and interdisciplinarity, is likely to reduce morbidity and unnecessary medical interventions. Ultimately, this study brings to light the importance of heightened awareness and individualized mental health care for those impacted by cardiac neurosis and promotes a more holistic, biopsychosocial model of diagnosis and treatment. An interdisciplinary team involving primary care, cardiology, and psychology, aligned with a biopsychosocial model, could improve care and reduce unnecessary medical interventions. Limitations such as self-report bias and limited generalizability must be acknowledged, and future longitudinal studies are needed.

## Appendices

**Table 7:** Cardiac Anxiety Questionnaire Reference: [6]

S.No	Items	Never	Rarely	Sometimes	Often	Always
1	I pay attention to my heartbeat	0	1	2	3	4
2	I avoid physical exertion	0	1	2	3	4
3	My racing heart wakes me up at night	0	1	2	3	4
4	Chest pain/discomfort wakes me up at night	0	1	2	3	4
5	I take it easy as much as possible	0	1	0	2	4
6	I check my pulse	0	1	2	3	4
7	I avoid exercise or other physical work	0	1	2	3	4
8	I can feel my heart in my chest	0	1	2	3	4
9	I avoid activities that make my heart beat faster	0	1	2	3	4
10	If tests come out normal, I still worry about my heart	0	1	2	3	4
11	I feel safe being around a hospital, physician, or other medical facility	0	1	2	3	4
12	I avoid activities that make me sweat	0	1	2	3	4
13	I worry that doctors do not believe my symptoms are real	0	1	2	3	4
When I have chest discomfort or when my heart is beating fast:						
14	I worry that I may have a heart attack	0	1	2	3	4
15	I have difficulty concentrating on anything else	0	1	2	3	4
16	I get frightened	0	1	2	3	4
17	I like to be checked out by a doctor	0	1	2	3	4
18	I tell my family or friends	0	1	2	3	4
